# Correction: Obesity and risk of female reproductive conditions: A Mendelian randomisation study

**DOI:** 10.1371/journal.pmed.1004095

**Published:** 2022-09-02

**Authors:** Samvida S. Venkatesh, Teresa Ferreira, Stefania Benonisdottir, Nilufer Rahmioglu, Christian M. Becker, Ingrid Granne, Krina T. Zondervan, Michael V. Holmes, Cecilia M. Lindgren, Laura B. L. Wittemans

Reference 45 is incorrect. The correct reference is:

Painter JN, Anderson CA, Nyholt DR, Macgregor S, Lin J, Lee SH, et al. Genome-wide association study identifies a locus at 7p15.2 associated with endometriosis. Nat Genet. 2011;43(1):51–4. pmid: 21151130
