# Supplementary material for: Obesity and risk of female reproductive conditions: A Mendelian randomisation study
Source: PLoS Med. 2022 Feb 1;19(2):e1003679. doi: 10.1371/journal.pmed.1003679 (PMC8806071; doi:10.1371/journal.pmed.1003679)
Supplement: S1 Checklist — (PDF) [file pmed.1003679.s001.pdf]

STROBE-MR checklist of recommended items to address in reports of Mendelian randomization studies[1]

Item No.	Section		Checklist item	Page No.	Relevant text from manuscript
1	**TITLE and ABSTRACT**		Indicate Mendelian randomization (MR) as the study's design in the title and/or the abstract if that is a main purpose of the study	Title	
	**INTRODUCTION**				
2	**Background**		Explain the scientific background and rationale for the reported study. What is the exposure? Is a potential causal relationship between exposure and outcome plausible? Justify why MR is a helpful method to address the study question	Introduction - paragraph 1	
3	**Objectives**		State specific objectives clearly, including pre-specified causal hypotheses (if any). State that MR is a method that, under specific assumptions, intends to estimate causal effects	Introduction - paragraph 2	
	**METHODS**				
4	**Study design and data sources**		Present key elements of the study design early in the article. Consider including a table listing sources of data for all phases of the study. For each data source contributing to the analysis, describe the following:		
		a)	Setting: Describe the study design and the underlying population, if possible. Describe the setting, locations, and relevant dates, including periods of recruitment, exposure, follow-up, and data collection, when available.	Methods, Two-sample Mendelian Randomisation - paragraphs 1 and 3; S1-S3 Tables	
		b)	Participants: Give the eligibility criteria, and the sources and methods of selection of participants. Report the sample size, and whether any power or sample size calculations were carried out prior to the main analysis	Methods, Two-sample Mendelian Randomisation - paragraphs 1, 3, and 4; S1-S3 Tables	
		c)	Describe measurement, quality control and selection of genetic variants	Methods, Two-sample Mendelian Randomisation - paragraph 2	
		d)	For each exposure, outcome, and other relevant variables, describe methods of assessment and diagnostic criteria for diseases	S1-S3 Tables	
		e)	Provide details of ethics committee approval and participant informed consent, if relevant	Methods, Research ethics - paragraph 1	
5	**Assumptions**		Explicitly state the three core IV assumptions for the main analysis (relevance, independence and exclusion restriction) as well assumptions for any additional or sensitivity analysis	Methods, Two-sample Mendelian Randomisation - paragraph 5	
6	**Statistical methods: main analysis**		Describe statistical methods and statistics used		

		a)	Describe how quantitative variables were handled in the analyses (i.e., scale, units, model)	Not applicable for two-sample MR, scales reported in sources described for each result
		b)	Describe how genetic variants were handled in the analyses and, if applicable, how their weights were selected	Methods, Two-sample Mendelian Randomisation - paragraph 2
		c)	Describe the MR estimator (e.g. two-stage least squares, Wald ratio) and related statistics. Detail the included covariates and, in case of two-sample MR, whether the same covariate set was used for adjustment in the two samples	Methods, Two-sample Mendelian Randomisation - paragraph 5
		d)	Explain how missing data were addressed	Not applicable for two-sample MR setting here
		e)	If applicable, indicate how multiple testing was addressed	Methods, Two-sample Mendelian Randomisation - paragraph 5
7	**Assessment of assumptions**		Describe any methods or prior knowledge used to assess the assumptions or justify their validity	Methods, Two-sample Mendelian Randomisation - paragraph 1
8	**Sensitivity analyses and additional analyses**		Describe any sensitivity analyses or additional analyses performed (e.g. comparison of effect estimates from different approaches, independent replication, bias analytic techniques, validation of instruments, simulations)	Methods, Two-sample Mendelian Randomisation - paragraphs 2, 3, and 5
9	**Software and pre-registration**			
		a)	Name statistical software and package(s), including version and settings used	Methods, Two-sample Mendelian Randomisation - paragraph 5
		b)	State whether the study protocol and details were pre-registered (as well as when and where)	Not applicable
	**RESULTS**			
10	**Descriptive data**			
		a)	Report the numbers of individuals at each stage of included studies and reasons for exclusion. Consider use of a flow diagram	No exclusions; S1-S3 Tables for sample size
		b)	Report summary statistics for phenotypic exposure(s), outcome(s), and other relevant variables (e.g. means, SDs, proportions)	Table 2; S7-S13 Tables
		c)	If the data sources include meta-analyses of previous studies, provide the assessments of heterogeneity across these studies	S2 Table
		d)	For two-sample MR:<br>i. Provide justification of the similarity of the genetic variant-exposure associations between the exposure and outcome samples	Results, Body fat distribution... - paragraph 4

			ii. Provide information on the number of individuals who overlap between the exposure and outcome studies	Results, Body fat distribution... - paragraph 4
11	**Main results**			
		a)	Report the associations between genetic variant and exposure, and between genetic variant and outcome, preferably on an interpretable scale	Too many for tables - dataset in Github linked in Data Availability
		b)	Report MR estimates of the relationship between exposure and outcome, and the measures of uncertainty from the MR analysis, on an interpretable scale, such as odds ratio or relative risk per SD difference	Results, Body fat distribution... - paragraphs 1 and 2; Table 2
		c)	If relevant, consider translating estimates of relative risk into absolute risk for a meaningful time period	Not applicable
		d)	Consider plots to visualize results (e.g. forest plot, scatterplot of associations between genetic variants and outcome versus between genetic variants and exposure)	Figures 2-3
12	**Assessment of assumptions**			
		a)	Report the assessment of the validity of the assumptions	Results, Body fat distribution... - paragraph 4
		b)	Report any additional statistics (e.g., assessments of heterogeneity across genetic variants, such as I^2, Q statistic or E-value)	S7-S13 Tables
13	**Sensitivity analyses and additional analyses**			
		a)	Report any sensitivity analyses to assess the robustness of the main results to violations of the assumptions	Results, Body fat distribution... - paragraph 4
		b)	Report results from other sensitivity analyses or additional analyses	Results, Body fat distribution... - paragraph 4
		c)	Report any assessment of direction of causal relationship (e.g., bidirectional MR)	Results, Body fat distribution... - paragraph 5
		d)	When relevant, report and compare with estimates from non-MR analyses	Results, Obesity traits are... - paragraph 1; Table 1
		e)	Consider additional plots to visualize results (e.g., leave-one-out analyses)	
	**DISCUSSION**			
14	**Key results**		Summarize key results with reference to study objectives	Discussion, paragraph 1
15	**Limitations**		Discuss limitations of the study, taking into account the validity of the IV assumptions, other sources of potential bias, and imprecision. Discuss both direction and magnitude of any potential bias and any efforts to address them	Throughout Discussion, particularly paragraph 10

16	**Interpretation**		
		a) Meaning: Give a cautious overall interpretation of results in the context of their limitations and in comparison with other studies	Throughout Discussion, particularly paragraphs 3, 7, and 8
		b) Mechanism: Discuss underlying biological mechanisms that could drive a potential causal relationship between the investigated exposure and the outcome, and whether the gene-environment equivalence assumption is reasonable. Use causal language carefully, clarifying that IV estimates may provide causal effects only under certain assumptions	Throughout Discussion, particularly paragraphs 7 and 8
		c) Clinical relevance: Discuss whether the results have clinical or public policy relevance, and to what extent they inform effect sizes of possible interventions	Discussion, paragraph 11
17	**Generalizability**	Discuss the generalizability of the study results (a) to other populations, (b) across other exposure periods/timings, and (c) across other levels of exposure	Discussion, paragraph 10
	**OTHER INFORMATION**		
18	**Funding**	Describe sources of funding and the role of funders in the present study and, if applicable, sources of funding for the databases and original study or studies on which the present study is based	Funding
19	**Data and data sharing**	Provide the data used to perform all analyses or report where and how the data can be accessed, and reference these sources in the article. Provide the statistical code needed to reproduce the results in the article, or report whether the code is publicly accessible and if so, where	Data Availability
20	**Conflicts of Interest**	All authors should declare all potential conflicts of interest	Competing Interests

This checklist is copyrighted by the Equator Network under the Creative Commons Attribution 3.0 Unported (CC BY 3.0) license.

1. Skrivankova VW, Richmond RC, Woolf BAR, et al. Strengthening the Reporting of Observational Studies in Epidemiology using Mendelian Randomisation (STROBE-MR): Explanation and Elaboration. BMJ. 2021;375:n2233.
