# Supplementary figures and images for: Obesity and risk of female reproductive conditions: A Mendelian randomisation study
Source: PLoS Med. 2022 Feb 1;19(2):e1003679. doi: 10.1371/journal.pmed.1003679 (PMC8806071; doi:10.1371/journal.pmed.1003679)

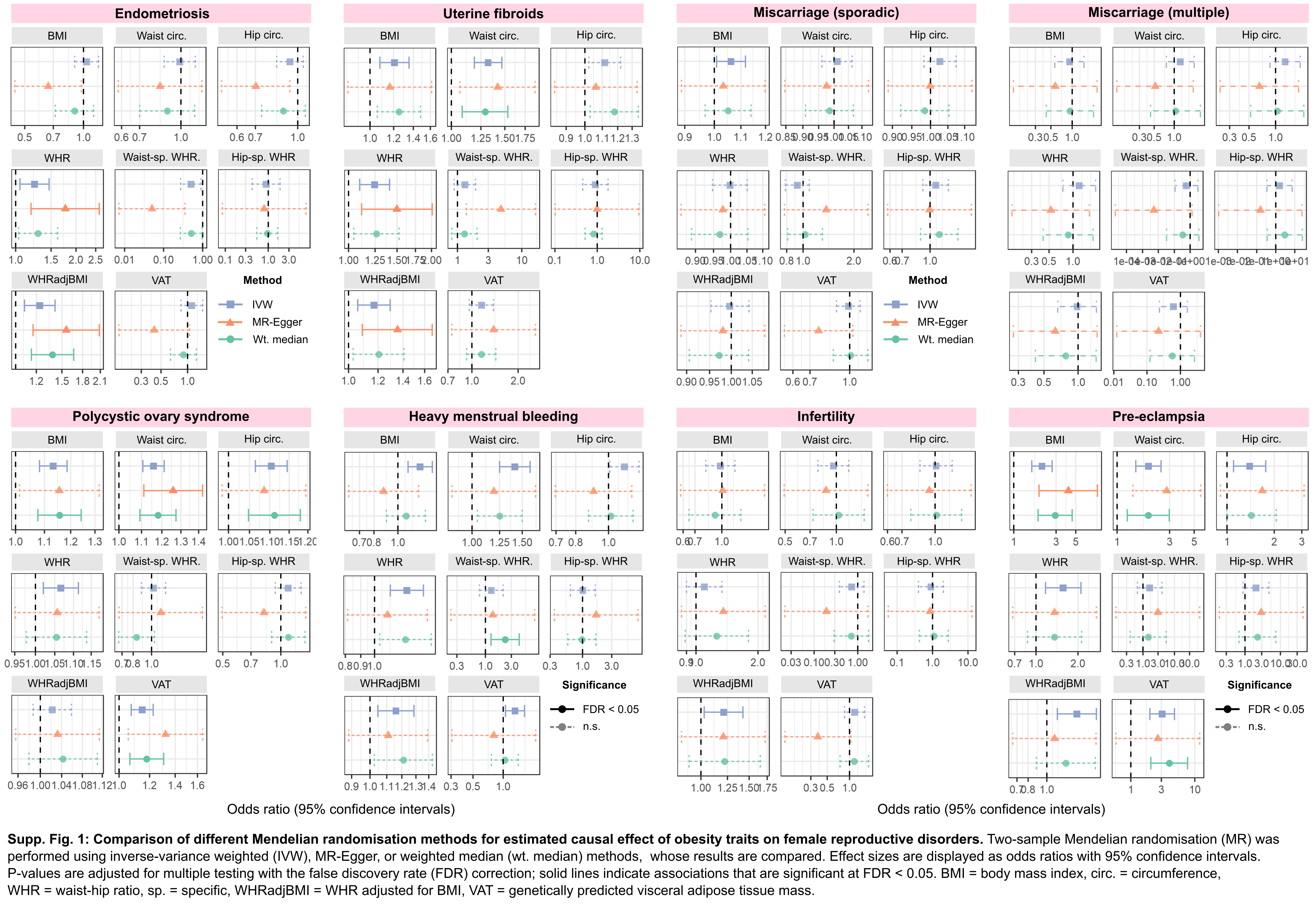

Supplement: S1 Fig — (TIF) [file pmed.1003679.s002.tif]

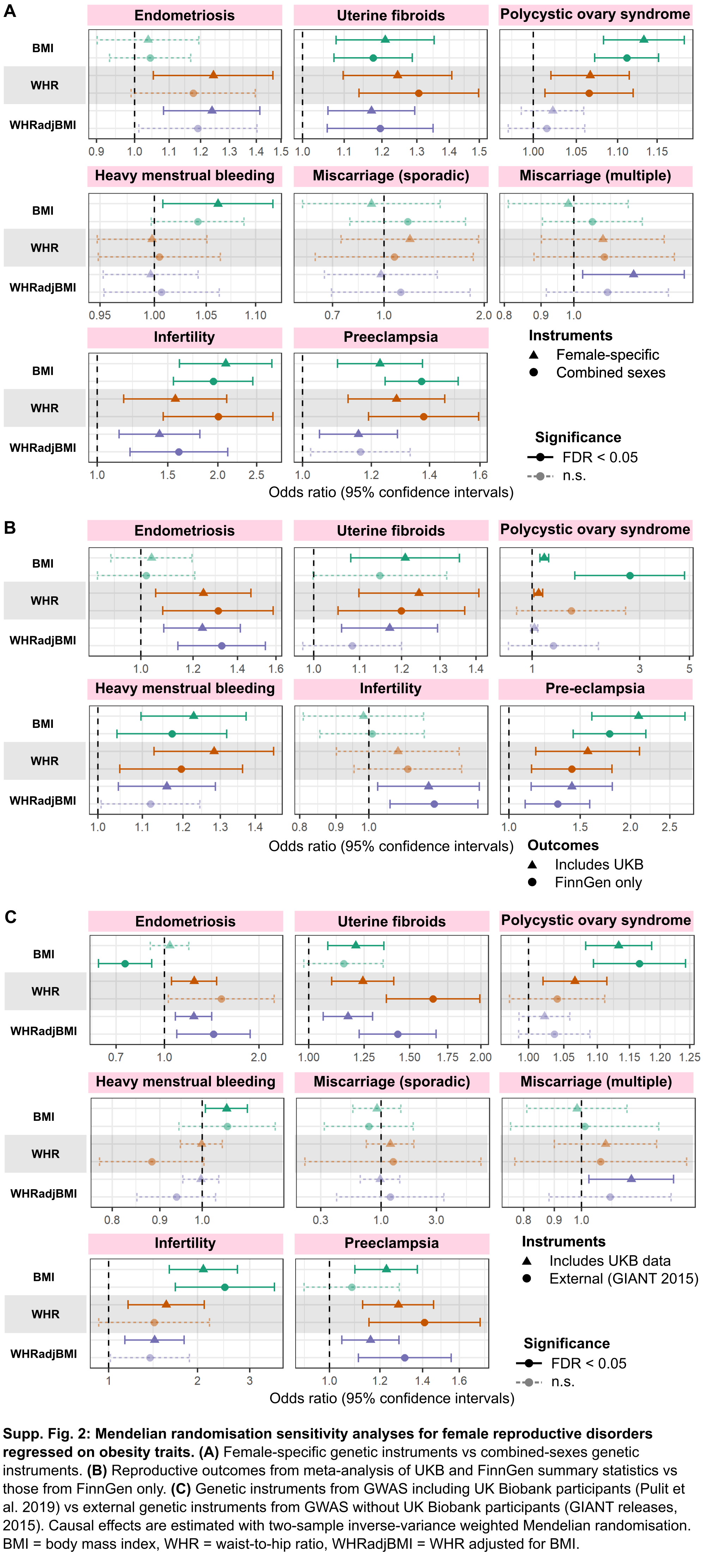

Supplement: S2 Fig — (A) Female-specific genetic instruments versus combined-sex genetic instruments. (B) Reproductive outcomes from meta-analysis of UK Biobank and FinnGen summary statistics versus FinnGen only. (C) Genetic instruments from GWASs including UK Biobank participants (Pulit et al. 2019 [34]) versus external genetic instruments from GWASs without UK Biobank participants (GIANT releases, 2015). (TIF) [file pmed.1003679.s003.tif]

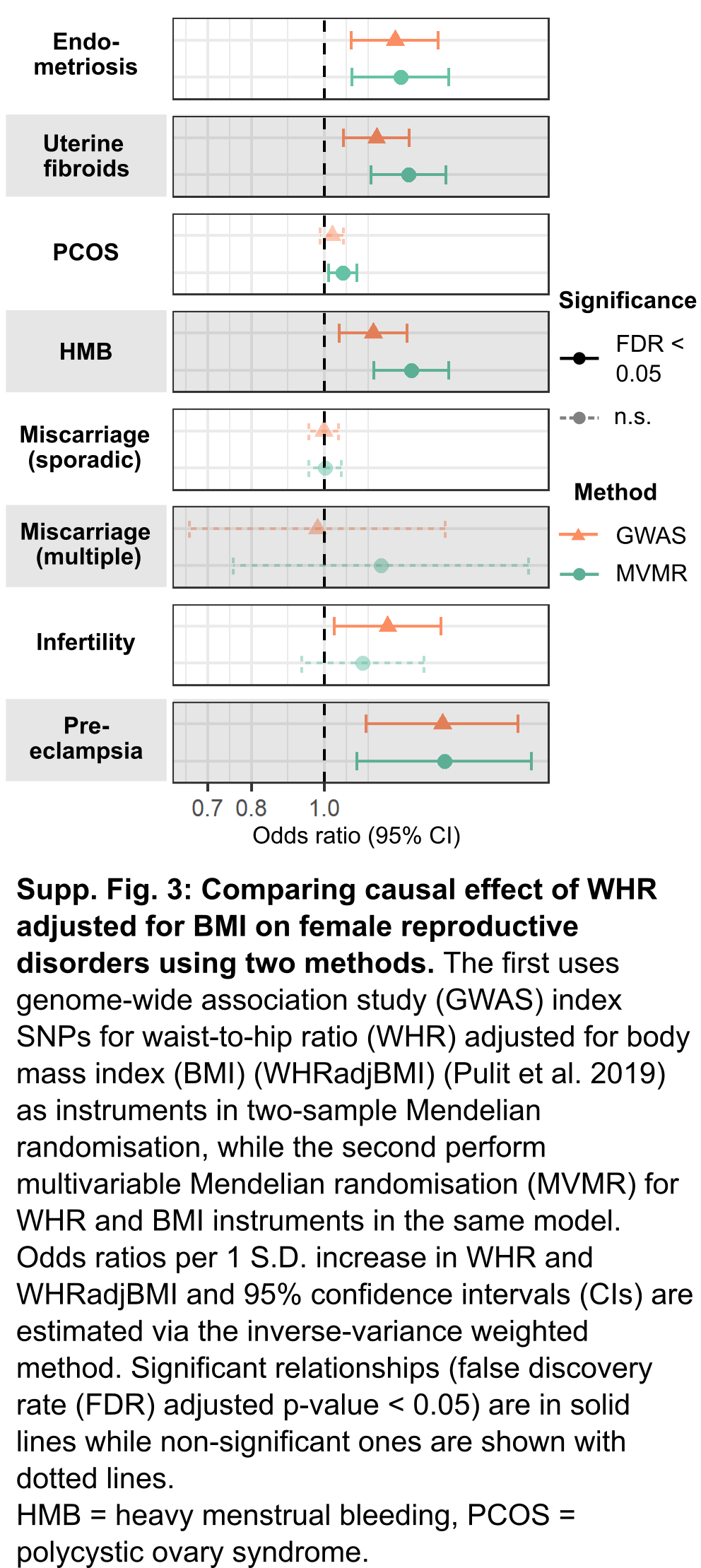

Supplement: S3 Fig — (TIF) [file pmed.1003679.s004.tif]

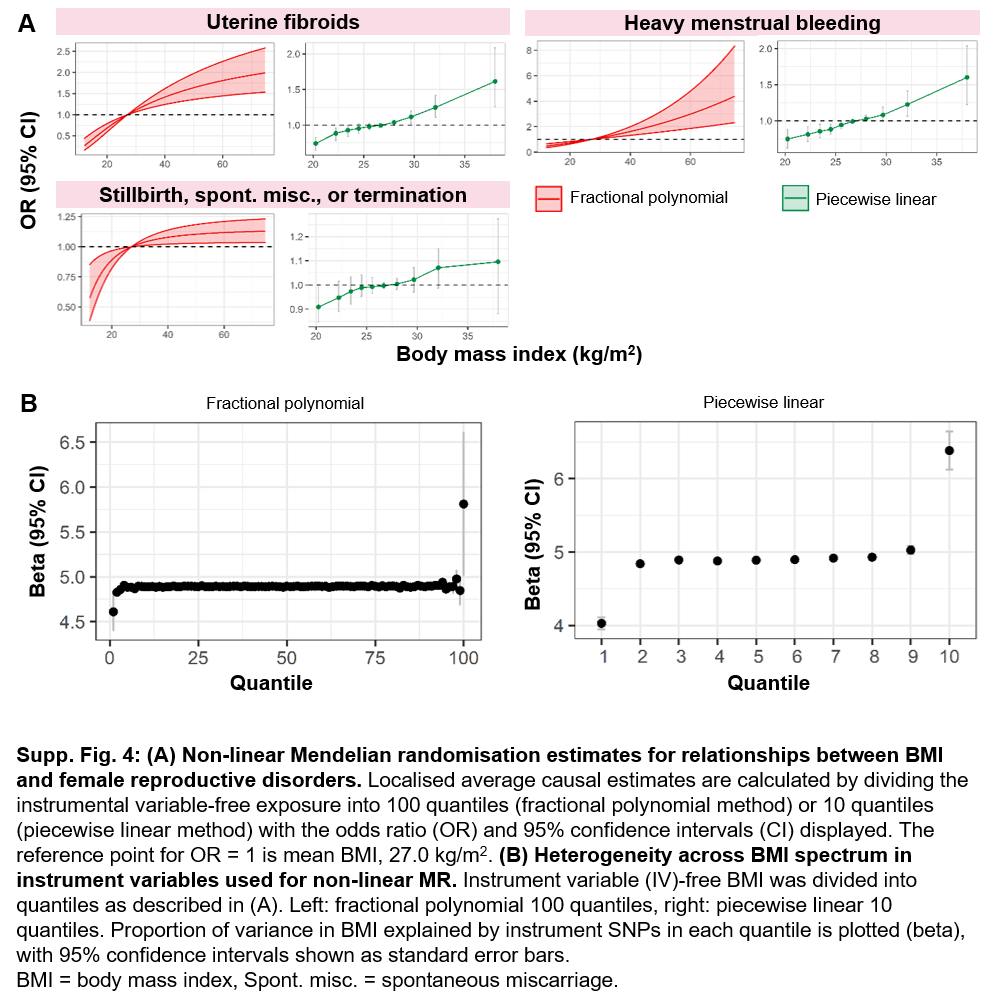

Supplement: S4 Fig — (A) Non-linear Mendelian randomisation estimates for relationships between BMI and female reproductive disorders. (B) Heterogeneity across BMI spectrum in instrument variables used for non-linear Mendelian randomisation. (TIF) [file pmed.1003679.s005.tif]

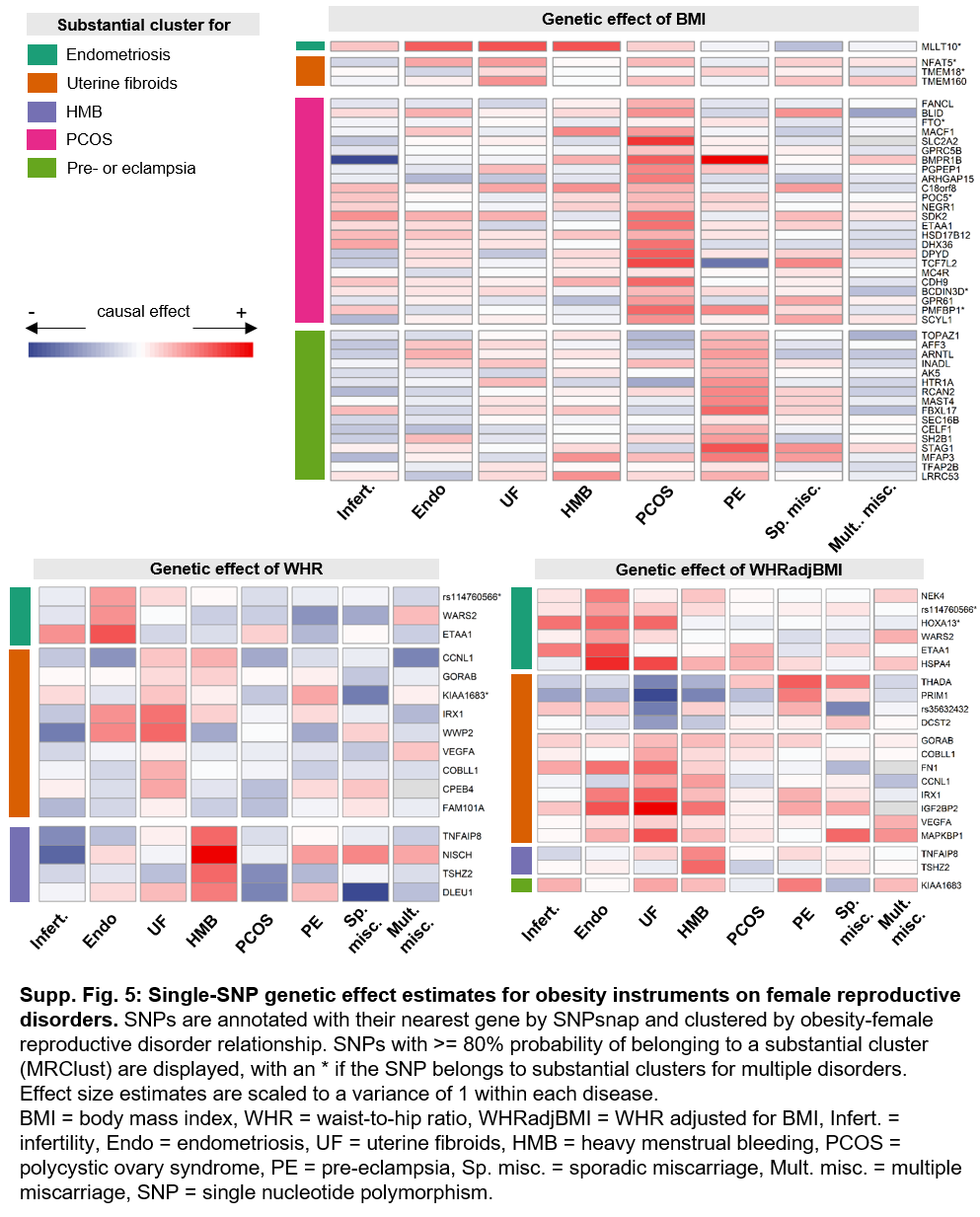

Supplement: S5 Fig — (TIF) [file pmed.1003679.s006.tif]
