# Supplementary material for: Obesity and risk of female reproductive conditions: A Mendelian randomisation study
Source: PLoS Med. 2022 Feb 1;19(2):e1003679. doi: 10.1371/journal.pmed.1003679 (PMC8806071; doi:10.1371/journal.pmed.1003679)
Supplement: Attachment — Submitted filename: Responses_to_Reviewers.docx [file pmed.1003679.s026.docx]

We thank the editors and reviewers for the time spent reviewing our paper. We appreciate the significant time commitment that was involved in providing these thoughtful comments. We are certain that the feedback has strengthened and refined our work. Following are our responses to each comment received. All line and page numbers referenced in the edits point to the clean revised manuscript.

### Request from the editors

#### General comments

Throughout the paper, please adapt reference call-outs to the following style: "... abnormal menstrual bleeding [1,2]..." (noting the absence of spaces within the square brackets).

Revised.

Throughout your manuscript, please substitute ‘causal effects’ with ‘casual associations’ or ‘causal relationships’
Substituted throughout the manuscript.

#### Competing Interests

Please confirm that all disclosed competing interests are outside the submitted work.
Confirmed and added to the competing interests statement.

#### Title

Please revise your title according to PLOS Medicine's style. We suggest “Obesity and risk of female reproductive disorders: A Mendelian randomisation study” or similar
Incorporated, new title: “Obesity and risk of female reproductive conditions: A Mendelian randomisation study”.

#### Abstract

##### Abstract Background

The final sentence should clearly state the study question.

The final sentence now reads: “Thus, we aimed to estimate observational and genetically predicted causal associations between obesity, metabolic hormones, and female reproductive disorders.”

##### Abstract Methods and Findings

Please provide brief demographic details of the study population (e.g. sex, age, ethnicity, etc)

Incorporated in lines 10-11 on page 2 of the clean revised manuscript.

Please include the number of individuals included in your study.
Incorporated on line 10, page 2.

Line 33 - please be more specific as to which conditions causal effect estimates of WHR and WHRadjBMI, but not BMI, were attenuated, and quantify those associations

We thank the editor for spotting this error in the original draft, which should read “Causal effect estimates of BMI, but not WHR and WHRadjBMI, were attenuated compared to their observational counterparts.” As we would have to name and quantify 9 associations (between 3 obesity traits and 3 reproductive disorders), and as this is not a major finding, we have removed this line from the Abstract.

Line 35 - please revise to causally associated with

Revised.

In the last sentence of the Abstract Methods and Findings section, please describe 2-3 of the main limitation(s) of the study's methodology.

The last sentence of the Abstract Methods and Findings now reads: “This study was limited in power by the low prevalence of female reproductive conditions among women in the UK Biobank, with little information on pre-diagnostic anthropometric traits, and by the susceptibility of MR estimates to genetic pleiotropy.”

##### Abstract Conclusions

Please begin your Abstract Conclusions with "In this study, we observed ..." or similar, to summarize the main findings from your study without overstating your conclusions.

The Abstract Conclusions now begin with: “We found that common indices of overall and central obesity increased risk of reproductive disorders to heterogenous extents in one of the first systematic, large-scale, genetics-based analysis of the aetiological relationships between obesity and female reproductive conditions.”

Please temper assertions of primacy by adding ‘to the best of our knowledge’ or similar

Revised.

Line 44 - please be more specific that metabolic hormones were only associated with pre-eclampsia, as this was the only association presented in the results.

As this was not central to the message, this line has been removed from the Abstract.

#### Author Summary

At this stage, we ask that you include a short, non-technical Author Summary of your research to make findings accessible to a wide audience that includes both scientists and non-scientists. The Author Summary should immediately follow the Abstract in your revised manuscript. This text is subject to editorial change and should be distinct from the scientific abstract. Please see our author guidelines for more information: <https://journals.plos.org/plosmedicine/s/revising-your-manuscript#loc-author-summary>

In the final bullet point of ‘What Do These Findings Mean?’, please describe the main limitations of the study in non-technical language.
Added in lines 31-56 on pages 3-4.

#### Introduction

Line 49 - please be more specific on the associations between BMI and gynaecological conditions i.e. higher vs lower
Unless we are mis-interpreting this comment, we do not think that this line, which begins with “BMI is associated with **increased** prevalence of gynaecological conditions…” (emphasis added), needs to repeat that associations are higher.

Line 73 - please be more specific re adverse pregnancy outcomes

This has been modified to say “hypertensive disorders of pregnancy” on line 88, page 6.

Line 78 - Please temper assertions of primacy by adding ‘to the best of our knowledge’ or similar

Revised.

#### Methods

Did your study have a prospective protocol or analysis plan? Please state this (either way) early in the Methods section. If a prospective analysis plan (from your funding proposal, IRB or other ethics committee submission, study protocol, or other planning document written before analyzing the data) was used in designing the study, please include the relevant prospectively written document with your revised manuscript as a Supporting Information file to be published alongside your study, and cite it in the Methods section. A legend for this file should be included at the end of your manuscript. If no such document exists, please make sure that the Methods section transparently describes when analyses were planned, and if/when reported analyses differed from those that were planned. Changes in the analysis-- including those made in response to peer review comments-- should be identified as such in the Methods section of the paper, with rationale. If a reported analysis was

performed based on an interesting but unanticipated pattern in the data, please be clear that the analysis was data-driven.

The following line has been added to the Methods: “The analyses upon which this paper is based were planned and designed for each of the sections separately; no overall prospective analysis plan was followed.”

Please ensure that the study is reported according to the STROBE guideline, and include the completed STROBE checklist as Supporting Information. Please add the following statement, or similar, to the Methods: "This study is reported as per the Strengthening the Reporting of Observational Studies in Epidemiology (STROBE) guideline (S1 Checklist)." The STROBE guideline can be found here: <http://www.equator-network.org/reporting-guidelines/strobe/> When completing the checklist, please use section and paragraph numbers, rather than page numbers which will likely no longer correspond to the appropriate sections after copy-editing.

Included.

#### Results

Please incorporate supplementary table A into the main paper as a table showing the baseline characteristics of the study population (to become Table 1) which should include the information included in paragraph 1 of your Methods section.

Added as Table 1.

Please report the number of participants early in your Results section.

Reported as: “All associations were estimated in 257,193 women of European ancestry in UKBB, with cases defined as in Table 1 and all non-cases as controls.” in the first paragraph of the Results.

For the ORs reported, please specify the comparison groups / reference point.

Included alongside the previous edit, i.e. “... with cases defined as in Table 1 and all non-cases as controls.”

For the observational component of your study, please provide the actual numbers of events for the outcomes in a table, not just ORs.

This is included in Table 1, which has been moved from the Supplement as requested.

Line 276 - please revise to ‘casually associated with’

Revised.

Line 325 - please clarify what is meant by ‘different effects’
This now states: “We found that leptin (50.2% of effect of BMI on pre-eclampsia), fasting insulin (27.7% - 36.6%), and ISI (19.1% - 50.1%) each mediated the total causal estimate of obesity traits on female reproductive disorders (Table 4)”.

#### Discussion

Lines 368 / 448 / 464 - Please temper assertions of primacy by adding ‘to the best of our knowledge’ or similar

Revised.

#### Figures

Please consider avoiding the use of red and green in order to make your figure more accessible to those with colour blindness.
We appreciate this reminder; all colour palettes have now been updated to colour-blind friendly palettes (specifically, palettes “Dark2” and “Set2” in the RColorBrewer package).

Please define all abbreviations used in the figure legend of each figure.
Incorporated in all figures, including supplemental figures.

Figure 1 - please clarify whether there is a part B missing

We thank the editor for noticing this error in the figure and legend, there is no part (B). This has now been updated.

Figure 4 - please revise to ‘estimated effect(s)’ or similar

Revised.

Figure 5a - please label the axes

Figure 5(A) contains UMAP plots, which are inherently unit-less and without reference values as they are simply projections of large matrices onto two-dimensional space. To the best of our knowledge, there is no label that can be assigned to these axes beyond “x” and “y”, which represent the two-dimensional space of projection.

Figure S1 - please use bolder colours in your plots as the bars and whiskers are difficult to see.

Revised.

#### Tables

Please define all abbreviations used in the table legend of each table, including supplementary tables.
Included in all tables and supplementary tables.

When a p value is given, please specify the statistical test used to determine it in the table legend.
Included in all tables and supplementary tables.

Table 2 - should the final column read P value (since most rows are adjusted for BMI / WHR / WHRadjBMI)?
This and the column headers in all tables have been revised to read “P-value” rather than “unadjusted P-value”, which we had meant to refer to P-values uncorrected for multiple testing. This has been clarified in the table footnotes instead.

#### Supplementary files

Please separate supplementary tables into S1 table, S2 table etc rather than S1 Table A / B etc. so that they can be individually accessed.
The tables have been separated and supplied as requested.

Please provide titles and legends for each individual table and figure in the Supporting Information.
This is now provided in the Supporting Information file.

### Comments from the reviewers

#### Reviewer #1

This is a very important work comprehensively examining the role of obesity in multiple female reproductive conditions. Observational estimates as well as genetically predicted effects of BMI and central adiposity were assessed. The Mendelian randomization techniques used represent the latest developments in the field. Complex patterns of causality between adiposity traits and reproductive conditions were observed. The Discussion accurately presents the findings with a view to future directions. Clarification of a few details will assist readers:

1. Participants in UKBB were age 40-69 at recruitment. Many of the conditions examined present at younger ages and may even resolve at older age (PCOS, for example). The conditions are identified based on ICD9 and ICD10 codes. If a condition happened in the past (or resolved in the past), there is a good chance that it would not be recorded in ICD codes. This may explain the extremely low rate of PCOS (0.3%). This limitation should be discussed in more detail, as it may explain the low rates of several conditions in addition to underdiagnosis.
   We agree that the low rate of reproductive conditions in UKBB may in part be due to the condition not being recorded in the electronic health records and/or being resolved many years/decades ago. However, we based our case definitions based on three distinct sources:
   a. The participants’ responses to a structured health questionnaire and interview about past and present health conditions, which included questions related to reproductive conditions.
   b. Primary care electronic health records which covers a period from 1938 at the earliest (or the first date at which the participant was registered with a GP practice that had an EHR system in place) until 2017 at the latest. The average age of UK Biobank participants at the first GP entry was 27.0 +/- 15.5 years, or 29.6 years before entry into the study.
   c. Secondary care in-patient electronic health records, starting from 1981 at the earliest.

Based on these 3 sources of information, we should be able to retrieve diagnoses patients received decades before entering UK Biobank. We however acknowledge that, even if a participant was diagnosed in the past, this may not have been captured in any of our 3 sources of information, for instance if the diagnostic code was not entered, if the diagnosis was made in a healthcare setting where the EHRs to which we have access were not (yet) implemented, or if the participant could during the baseline assessment not recall ever having received the diagnosis. We have included this limitation now more extensively in the Discussion, lines 536-542.

2. Line 123: P < 5E-9 is quoted. However, 5E-8 is much more commonly used as the genome-wide significance level. Please correct or explain.
   This is as reported in the instrument GWAS source, Pulit et al. 2018 [ref.1] to account for the denser imputation data. This text has been added to line 145 on page 11. In recent years, there has been a tendency to lower the genome-wide significance threshold to p<5E-9, as imputation panels have become denser and therefore the number of independent tests greater.
3. Line 127: "waist-specific and hip-specific WHR" is unclear. What was meant does not become clear until reading results several pages later.
   This has now been clarified as “WHR instruments with specific effects on waist but not hip circumference and vice-versa”.
4. On line 99, the UKBB numbers for PCOS are given as 746 cases and 256,447 controls. However, line 151-152 describes a UKBB GWAS of 11,186 cases and 273,812 controls. Please reconcile the numbers.

On line 99 in the original manuscript, we had described the 746 PCOS cases and 256,447 controls which were included in the observational analyses conducted based on **UK Biobank participants only**. For the genetic analyses, which were described in lines 151-152, we combined GWAS data from multiple sources, which included UK Biobank, as well as GWAS summary stats released by FinnGen and the PCOS consortium, which in total amounted to 11,186 cases and 273,812 controls. To clarify this further to the reader we have now added **S3 Table**, which outlines the datasets that were included in the meta-GWAS for PCOS.

5. As there are multiple methods of calculating F statistic, the method used here should be given.

This has now been specified as “mean β^2^/σ^2^ over all SNPs in the instrument”.

6. The Walford GWAS for Stumvoll insulin sensitivity index found very few signals at genome-wide significance. What are the implications of that for the current study?
   The reviewer is correct in noting that there are only 5 index SNPs identified by the Walford et al. GWAS for Stumvoll insulin sensitivity index. However, the instrument F-statistic (mean β^2^/σ^2^ over all SNPs in the instrument) is 26.6 (**S1 Table**), while weak instrument bias is conventionally noted at F < 10 [ref.2]. Therefore, despite the sparsity of genome-wide signals, the associations are strong enough to lead us to the conclusion that our MR results are robust.
7. Figure 1 highlights instances where "non-linear models fit the data better than linear models" whereas line 289 states that "no non-linear models explained the causal effects of BMI … better than linear MR models." Please clarify.
   This appears to be a misunderstanding, as Figure 1 displays observational associations where non-linear models better fit the data; the line referenced is describing genetic associations, i.e. non-linear Mendelian randomization models.
8. Effects of three potential mediators (leptin, FI, IR) were examined individually. Is there an opportunity here to examine them jointly?
   We thank the reviewer for this suggestion. In general, when more exposures (variables) are incorporated in MVMR, the lower is the power to detect true causal effects [ref.3]. Indeed, when we attempted to jointly adjust for the effects of leptin and FI or leptin and ISI with obesity traits, we did not find any significant effects of any of the examined variables, likely due to reduced power.
9. "junk cluster" seems to apply a value judgement to certain variants. However, if that is the accepted term for this technique, it can be used.
   We accept and share this sentiment, however this is the referenced terminology in the MR-Clust technique to differentiate among “null” (no effect) or “substantial” SNPs and those that do not belong to any cluster, with the latter being termed “junk” [ref.4].
10. Metformin should not be referred to as "first line treatment' (line 486).
    Removed.

#### Reviewer #2

The authors present a very interesting and detailed manuscript exploring the role of obesity-related traits in relation to a number of reproductive and menstrual conditions. They perform both observational and Mendelian randomization analysis, including the use of bi-directional MR and two-step approaches to investigate mediating mechanisms. Further, they look at the use of genetic variants to provide insights into the clustering of reproductive conditions in relation to adiposity.

1. In the Introduction, more specific details from previous studies assessing the relationship between obesity and reproductive traits would be useful, including effect estimates and the relative strengths and limitations of these previous approaches in order to highlight motivation for the present analysis.

While we appreciate and share the sentiment expressed in this comment, we believe that adding specific effect estimates to comprehensively compare previous studies is challenging and out of scope for this manuscript due to the following reasons: (1) effect estimates from different study types, i.e. case-control studies, population-based studies, and prospective cohorts, with diverse selection of cases and populations, cannot be compared in a straightforward manner, and (2) without a systematic review for each obesity trait - reproductive disease relationship (of which there are 24+), we would inevitably miss some previously published estimates.

Instead, we have succinctly added the following lines to the Introduction: “Previous estimates of the associations of obesity with reproductive conditions have primarily been based on observational study designs including case-control studies (McCann et al., Sun et al., Lashen et al.), cross-sectional studies in randomly selected women (Wei et al.), or only in women with obesity (Douchi et al.). While the direction of effect is largely consistent across studies, the heterogeneity in selection of cases, controls, and populations observed in these studies is reflected in heterogeneity in the effect estimates.”

2. After the statement "Women with advance-stage endometriosis have lower BMI than those with minimal disease, and the inverse BMI-endometriosis association is stronger in women with infertility", the authors should specifically highlight potential issues of reverse causation and selection bias here.

This statement is drawn from the results of a large prospective cohort study (NHSII) [ref.5], which is designed to minimise reverse causation and selection bias.

3. In the Methods, there are issues with using the GWAS for WHRadjBMI, which may introduce bias into the MR analysis: <http://dx.doi.org/10.1093/ije/dyaa266> . Instead, could the authors perform MVMR with both WHR and BMI in same model?
   We have added a sensitivity analysis using MVMR with WHR and BMI in the same model, which shows that results are not substantially different when using the different methods (**S11 Table** and **S3 Figure**). For all outcomes, the 95% CIs produced by WHRadjBMI SNPs and MVMR for WHR and BMI overlap. A succinct explanation of this additional analysis has been added to the Methods, lines 162-164 on page 12 and Results, lines 348-351 on pages 22-23.
4. There are issue of temporality with the adiposity measures often assessed after the diagnosis of reproductive conditions in the UK Biobank. While this is likely to be less of a thread in the MR analysis, this does have implications for the observational analysis. Could the authors run a sensitivity analysis using reported adiposity at an earlier timepoint? For example, in UK Biobank the participants were asked about their comparative body size at age 10.
   This analysis has now been added, and we have added the following lines to the Results: “We additionally found that being comparatively plumper than average at age 10 years, as self-reported, was associated with increased prevalence of endometriosis (OR = 1.12, P = 2.67E-04), heavy menstrual bleeding (OR = 1.18, P = 3.43E-15), PCOS (OR = 1.92, P = 1.59E-13), and uterine fibroids (OR = 1.07, P = 1.03E-03) (**S6 Table**). However, being thinner than average in early life was also associated with increased prevalence of endometriosis (OR = 1.21, P = 1.20E-13), heavy menstrual bleeding (OR = 1.10, P = 7.03E-08, and miscarriage (OR = 1.09, P = 4.03E-18).”
   We have also added the following lines to the Discussion: “Indeed, we noted that being thinner than average at age 10 years posed a higher risk for development of endometriosis than did being plumper, although both were associated with higher prevalence of disease compared to those who reported average adiposity in early life.”
5. The authors mention weighting the GRS but there are issues of introducing bias due to winner's curse and sample overlap when doing this (as mentioned). Would it be possible to run an MR sensitivity analysis where the SNPs are weighted by the effects seen in FinnGen?
   We acknowledge the potential bias mentioned; FinnGen only provides summary statistics for disease phenotype endpoints, and therefore does not have anthropometric measures of BMI, WHR, or WHRadjBMI represented. Instead, we addressed this by using instruments from the 2015 releases of GIANT summary statistics that do not include UK Biobank data [refs.6,7]. The results of these sensitivity analyses are now presented in **S9 Table** and **S2 Figure (C)** and briefly reported in the Methods, lines 160-162 on page 12, and Results, lines 345-346 on page 22.
6. The authors present power calculations but it is unclear how the results of a-priori power calculation were used to inform on whether further analyses should be conducted.

Our main motivation to conduct power calculations for the MR analyses was to aid in interpretation of our findings, and not to decide whether the analyses should be run in the first place. More specifically, when the MR analyses do not reveal a significant effect, we use the power calculation to estimate how likely it is that a true but small causal effect which we cannot pick up due to lack of power may still exist.

7. The authors mention that the IVW method was selected as the preferred method based on the Rucker framework but they should still acknowledge susceptibility of this test to horizontal pleiotropy
   This is now incorporated on line 196 on page 13.
8. Did the authors consider the use of Steiger filtering when assessing the bi-directional relationships?
   We did apply Steiger filtering to the index SNPs for endometriosis, PCOS, and uterine fibroids, resulting in 15/16, 13/14, and 23/29 SNPs respectively that survive filtering with substantially larger R2 with exposure than with outcome. The results are therefore not substantially changed and instrument strength still remains too weak (F-statistics < 10) to draw robust conclusions.
9. The authors should consider comparing the results of the two-step MR product of co-efficients estimates with those obtained from MVMR and difference method for estimating mediation.
   We attempted to compare the results of the two-step MR product of coefficients with that of the difference of estimates method; however, the wide confidence intervals of each estimate and the resulting propagation of uncertainty make meaningful comparison difficult. We find that the 95% CI bounds of proportion of effect mediated are between 0 - 100% in many cases.
10. More details on the use of UMAP for SNP clustering would be useful.
    We have now elaborated on how we utilised UMAP to cluster diseases by the effect of SNPs in obesity instruments as follows: “Each 8-by-M matrix of SNP effect estimates (for 8 female reproductive outcomes, where M is 281 for BMI, 203 for WHR, and 266 for WHRadjBMI) was reduced to a two-dimensional representation while maintaining as close a topological relationship between the 8 reproductive outcomes as possible, as measured by cross entropy (McInnes et al. 2018).” [ref.8]
11. Could the authors provide more information on the distinction between the non-linear observational models (generalized additive models and fractional polynomials) and how these approaches compare with the non-linear MR?
    Fractional polynomial regression is an extension of linear regression allowing for linear, quadratic, cubic, and *n*th degree terms in the explanatory variable; generalised additive models are extensions of generalised linear models that allow for smooth functions, such as splines, of the explanatory variable. We have added the following line to the Methods: “The latter allows a greater degree of flexibility in modelling curves that cannot be represented by polynomials of the *n*th degree, however their terms are more complex and thus less immediately interpretable (Jones & Wrigley 1995).” [ref.9] Non-linear Mendelian randomisation, on the other hand, divides the population into strata based on the instrument-free exposure (explanatory variable) and calculates a localised average causal effect (LACE) in each stratum. The LACE estimates are combined in either a fractional polynomial model (via meta-regression of LACE estimates), or in a piecewise linear model (via gradient of LACE estimates) [ref.10].
12. How were AICs compared to determine best fit of non-linear models? Was a test for difference in AIC performed?
    As is standard in the field, linear and non-linear models were compared by the difference in AIC (Δ = AIC_linear_ - AIC_non-linear_), following the guidelines suggested in [ref.11], i.e. Δ < 2 indicated little to no support for the more complex non-linear model, while Δ > 10 indicated very strong support for the non-linear model. This is now specified on line 139 on page 11.
13. In Table 1, the estimates for miscarriage (multiple consecutive) are missing for the observational analysis. Include a footnote to indicate why this is the case.
    We thank the reviewer for noticing this, a footnote indicating that data on multiple consecutive miscarriage was not available in UK Biobank for the observational analysis has now been included.
14. Did the authors perform a test for difference to compare the observational and MR aresults?
    The P-values from Z-tests for heterogeneity have now been added to the text comparing the difference between observational and MR results. The lines now read: “The causal estimates of WHR and WHRadjBMI on several reproductive disorders were higher than their observational counterparts (heterogeneity P-het < 0.0151 for 6/14 associations).” and “MR estimates of associations between BMI and endometriosis (OR = 1.04, P = 0.606), and PCOS (OR = 1.13, P = 7.60E-08) were much attenuated compared to observational results (P-het = 2.82E-30).”
15. The authors report that MR estimates of association between BMI and HMB, endometriosis and PCOS were attenuated compared with observational. However, for both HMB and PCOS there is a small but very precise effect from the MR (e.g. OR 1.01 for HMB). It is unusually to get such a precise MR effect and CIs appear to be smaller here than the corresponding observational analysis. Could the authors explain this?
    Upon double-checking our calculations for the HMB MR, we noticed that the betas reported in the summary statistics had not been properly converted to log odds and have therefore corrected that error. All results (text, tables, and figures) have been updated to reflect that correction, which has led to small changes in the reported effect size estimates, but note that these changes do not alter the main message or direction of effect.

Furthermore, in earlier MR papers, observational analyses were often more powered than the MR analyses, given that the latter often relied on much smaller and less powered GWAS and thus weaker and fewer genetic instruments than we have available today. In particular for obesity traits, current GWAS are very well powered and have led to the identification of 316-670 genetic instruments for WHR, WHRadjBMI and BMI, which drastically improves the power of MR analyses.

16. The authors should compare the IVW results with those from more pleiotropy-robust methods (shown in from Figure S1) in the text.

We have now included the following sentence: “We prioritised MR results based on the IVW method over less powered MR-Egger and weighted median methods, as there was no evidence for directional pleiotropy (MR-Egger horizontal pleiotropy P > 0.0547) and the ratio of Cochran’s Q’ (Egger) to Q (IVW) was > 0.876 (**S7 Table**). For the 24/64 analyses for which IVW indicated a significant effect, the effect estimate of the other methods was either directionally consistent (13/24) or non-significant (11/24), but never opposite (**S1 Figure**).”

17. The authors claim that non-linear models explained the observational data better than linear models, but this was not the case for the MR analysis. As well as indicating potential low power in the MR to detect non-linear relationships, could the authors speculate on any other reasons for this?

Given the relatively low case numbers in UK Biobank and the fact that the non-linear MR analyses required us to bin cases based on their BMI category, which even further reduced the number of cases included in each sub-analysis, we are of the opinion that the most likely explanation for the apparent discrepancy between our observational and genetic findings is the lack of power to conduct non-linear MR analyses. Another potential contributing factor could be that our current GWAS approach to finding genetic instruments for BMI is inadequate to estimate causal effects of a BMI increase in a specific range of the total BMI spectrum. While the currently available GWAS on BMI focus on identifying the genetic determinants of BMI across the full BMI distribution, it is possible that different genetic factors explain variations in BMI among those with a BMI between 20 and 25 compared to those with a BMI between 40 and 45. There is currently no evidence for this, but if it were indeed the case, then the genetic instruments used in the present analyses would be sub-optimal to discover non-linear causal effects. We have now included this in the Discussion (lines 477-481 on page 31).

18. Unless I missed it, I was unclear why non-linear MR was only run for UF, HMB and stillbirth/miscarriage. Was this indicated based on power calculations?
    This was mentioned in the Methods of the original manuscript, lines 192-194 on page 9, and in the revised manuscript lines 221-223, page 15, as: “Outcomes were restricted to female reproductive disorders with prevalence > 5% in UKBB (i.e. HMB, miscarriage, and UF) to maintain sufficient sample sizes in each quantile to estimate localised average causal estimates”.
19. Unless I missed it, I was unclear why reverse MR was only run for endometriosis, PCOS and UF? Was this indicated based on power calculations?
    This was mentioned in the Results of the original manuscript, lines 306-308 on page 17 and in the revised manuscript lines 355-357 on page 23, as: “We were limited in assessing reverse causality of other female reproductive conditions on obesity traits by the lack of index SNPs in large-scale publicly available GWAS summary statistics.”
20. Could the authors consider the use of weak instrument correction methods for the reverse MR?
    To the best of our knowledge, the two weak instrument correction methods developed for two-sample MR, i.e. Robust Adjusted Profile Score (MR-RAPS) [ref.12] and radial MR [ref.13] are both unsuitable here as they only have the power to detect true causal effects if there are hundreds of weak instruments; we have only a few SNPs (14-29) in each instrument.
21. The authors perform two-step analysis considering leptin, fasting insulin and insulin sensitivity as mediators. Was there any evidence that these factors in turn influence adiposity (i.e. may also be potential confounders?) This will have implications for the estimation of the mediated effect.
    We assessed the potential for reverse causality with reciprocal MR with instruments for leptin, FI, and IR regressed on BMI, WHR, and WHRadjBMI as outcomes, and found no reverse association (**S19 Table**). We have now mentioned this in lines 371-372 on pages 23-24.
22. It is interesting that leptin, fasting insulin and ISI were associated with risk of PE after adjustment for BMI, but not in unadjusted analysis. Why might this be?

The univariable effects of leptin, FI, and ISI rely on a few genetic variants that reach genome-wide significance; however, when BMI instruments are included in the multivariable analysis for mutual adjustment, there are likely several BMI variants with nominally significant effects on leptin, FI, and ISI that did not reach genome-wide significance (P < 10E-8). Therefore, a different set of variants is being compared in the univariable and multivariable analyses. We have added a footnote to **Table 3** to clarify this further.

23. The authors should explain the likely reason for the high proportion of women reporting "unsure" for menopause - e.g. having had a hysterectomy.
    This has now been added on lines 464-465 on page 31.
24. There is a detailed discussion contextualizing findings but more about the relative strengths/limitations of MR (including assumptions) and other genetic approaches used in this context would be useful.
    We have now included a discussion on MR methods used in this investigation, their limitations, and interpretations of the estimates to the Discussion, lines 428-435 on page 29.

#### Reviewer #3

I would like to congratulate the authors on a very comprehensive and convincing study on the effect of obesity on female reproductive conditions. Venkatesh et al use both

observational and genetic study design to triangulate evidence and use a wide range of methodology ranging from logistic regression to Mendelian randomization (MR), and also include advanced methods like for example nonlinear models and mediation analysis. Also I would like to thank the authors for providing the R-code as a github repository; this is much appreciated and a great example for reproducible science.

My comments are mainly focused on the wording, in particular the careless use of the "causal" word, and the results from the mediation analysis.

##### Major comments

1. Interpretation of MR estimate: The authors interpret the MR effect estimate throughout the manuscript as "causal". MR is a causal inference method, but MR relies on strong and mainly untestable assumptions. Consequently, I would urge the authors to adapt a more cautious interpretation of the MR effect estimate in the Abstract, Methods, and Results section in line with the MR guidelines [1]. Please see a nuanced discussion how MR estimates should be interpreted in Section 9 of the guidelines [1].

For example, instead of distinguishing between observational and "causal" associations, the authors may distinguish between observational and "genetic" associations. Instead of "Causal effect estimates" the authors may use the term "MR effect estimates". Finally, phrases like "Genetically predicted visceral adipose tissue mass was causal for" should be changed into "Genetically predicted visceral adipose tissue mass was associated with".

Following the MR guidelines [1] the authors could then add a paragraph discussing the "causal interpretation" in the light of the plausibility of the instrumental variable assumptions, the concordance of estimates from different methods and different analytical approaches, the results from sensitivity and supplementary analyses. Here, the authors can make a very strong point given the extensive analysis presented in the manuscript that these findings are indeed likely to be causal.
We appreciate this comment and have tempered the language throughout our manuscript to read “genetically predicted effect”, “genetic effect”, or “genetically predicted causal estimate” where appropriate. We have also included a paragraph in the Discussion (lines 428-435 on page 29) that discusses more thoroughly the interpretation of MR estimates as causal.

2. Mediation analysis: The results of the multivariable MR (Table 2) describing the effect of leptin, fasting insulin and insulin sensitivity on pre-eclampsia are surprising. Could the authors please comment on why there is no significant effect in the unadjusted univariable MR model (which instruments are used here?) and there is a highly significant effect in a multivariable MR model when adjusting for BMI (are the same instruments used as in the univariable model or does this analysis include BMI instruments as well)? When interpreting multivariable MR results in terms of total and direct effect [2], how can the total effect of an exposure be Null, while there is a strong direct effect? Is there a biological explanation for this?
   As the reviewer notes, the univariable effects of leptin, FI, and ISI rely on a few genetic variants that reach genome-wide significance; however, when BMI instruments are included in the multivariable analysis for mutual adjustment, there are likely several BMI variants with nominally significant effects on leptin, FI, and ISI that did not reach genome-wide significance (P < 10E-8). Therefore, a different set of variants is being compared in the univariable and multivariable analyses. We have added a footnote to **Table 3** to clarify this.
3. Ruecker's framework: To the best of my knowledge Ruecker's framework (please note Rücker or Ruecker, not Rucker) can be used to distinguish if the IVW or MR-Egger method provides the better model fit. It cannot be used to compare results from the weighted median MR, because the Q-statistic is not defined in the median MR model. Please correct on page 8, line 163 and remove weighted median from your list. No changes need to be made to the results or Table F in the Supplementary. It would be perfectly appropriate to consider IVW being the most powerful method as the main method and MR-Egger and the weighted median as sensitivity analysis providing more pleiotropy-robust inference.
   We have made these corrections in lines 192-194, 197 on pages 13-14. The spelling error has been corrected throughout the manuscript.

##### Minor comments

1. FDR adjustment: When performing FDR correction, for how many tests did you adjust? The number of exposures, the number of outcomes, or the number of exposures times the number of outcomes? Please clarify. Which method did you use for FDR correction? Please provide a citation.
   Multiple testing correction was applied to the number of exposures x number of outcomes, the exact numbers have now been added in lines 302 (page 20), 334 (page 22), 373 (page 24) in the Results section as well as lines 121 (page 7) and 198 (page 14) in Methods. We used the Benjamini-Hochberg method for FDR adjustment, the citation for which has been added in line 123 (page 7).
2. Page 6, line 123, genome-wide significance, why did you use 5E-9 and not 5E-8?
   This is as reported in the instrument GWAS source, Pulit et al. 2018 [ref.1] to account for the denser imputation data. This text has been added to line 145 (page 11). In recent years, there has been a tendency to lower the genome-wide significance threshold to p<5E-9, as imputation panels have become denser and therefore the number of independent tests greater.
3. Page 11, line 223, please introduce the abbreviation UMAP.
   The abbreviation UMAP has now been expanded as “Uniform Manifold Approximation and Projection”.
4. Page 11, line 227, MR-Clust v0.1.0 R package (56). Is citation (56) to Pers et al correct? Or should this rather be citation (57) Foley et al?
   The citation has been corrected in line 260 (page 16).

##### References from reviewer #3

1. Burgess, S. et al. Guidelines for performing Mendelian randomization investigations [version 2; peer review: 2 approved]. Wellcome Open Res. 4, (2020).
2. Burgess, S., Daniel, R. M., Butterworth, A. S., Thompson, S. G. & the EPIC-InterAct Consortium. Network Mendelian randomization: using genetic variants as instrumental variables to investigate mediation in causal pathways. Int. J. Epidemiol. 44, 484-495 (2015).

**References in rebuttal**

1. Pulit SL, Stoneman C, Morris AP, Wood AR, Glastonbury CA, Tyrrell J, et al. Meta-analysis of genome-wide association studies for body fat distribution in 694 649 individuals of European ancestry. Hum Mol Genet. 2019;28(1):166-74.
2. Bound J, Jaeger D, Baker RM. Problems with instrumental variables estimation when the correlation between the instruments and the endogenous explanatory variable is weak. Journal of American Statistical Association. 1995;90:443-50.
3. Sanderson E, Spiller W, Bowden J. Testing and Correcting for Weak and Pleiotropic Instruments in Two-Sample Multivariable Mendelian Randomisation. bioRxiv. 2020.04.02.021980
4. Foley CN, Mason AM, Kirk PDW, Burgess S. MR-Clust: clustering of genetic variants in Mendelian randomization with similar causal estimates. Bioinformatics. 2021;37(4):531-41.
5. Belanger CF, Hennekens CH, Rosner B, Speizer FE. The nurses’ health study. The American Hournal of Nursing. 1978;78(6):1039-40.
6. Locke AE, Kahali B, Berndt SI, Justice AE, Pers TH, Day FR, et al. Genetic studies of body mass index yield new insights for obesity biology. Nature. 2015;518:197-206.
7. Shungin D, Winkler TW, Croteau-Chonka DC, Ferreira T, Locke AE, Mägi R, et al. New genetic loci link adipose and insulin biology to body fat distribution. Nature. 2015;518:187-196.
8. McInnes L, Healy J, Melville J. UMAP: Uniform Manifold Approximation and Projection for Dimension Reduction. arXiv:1802.03426.
9. Jones K, Wrigley N. Generalized Additive Models, Graphical Diagnostics, and Logistic Regression. Geographical Analysis. 1995;27:1-18.
10. Staley JR, Burgess S. Semiparametric methods for estimation of a nonlinear exposure-outcome relationship using instrumental variables with application to Mendelian randomization. Genetic Epidemiology. 2017;41:341-352.
11. Burnham KP, Anderson DR. Multimodel Inference: Understanding AIC and BIC in Model Selection. Sociological Methods Research. 2004;33:261-304.
12. Zhao Q WJ, Hemani G, Bowden J, Small DS. Statistical inference in two-sample summary-data Mendelian randomization using robust adjusted profile score. arXiv 2018.
13. Bowden, J., et al., Improving the visualization, interpretation and analysis of two-sample summary data Mendelian randomization via the Radial plot and Radial regression. International Journal of Epidemiology. 2018;47(4):1264-1278.
