# Supplementary material for: Obesity and risk of female reproductive conditions: A Mendelian randomisation study
Source: PLoS Med. 2022 Feb 1;19(2):e1003679. doi: 10.1371/journal.pmed.1003679 (PMC8806071; doi:10.1371/journal.pmed.1003679)
Supplement: Attachment — Submitted filename: Response_to_editor_requests.docx [file pmed.1003679.s027.docx]

**Requests from Editors:**

1. Data availability statement: Please include the sentence from the Methods at line 273-274 with the link for the analysis scripts. “All scripts used in analyses are deposited at [https://github.com/lindgrengroup/obesity_femrepr_MR”](https://github.com/lindgrengroup/obesity_femrepr_MR%E2%80%9D)

Included.

2. Title: Please change to “A Mendelian randomization study” as the subtitle.
Changed.

3. Abstract: Lines 12-18: Please quantify the main results presented in the Abstract with ranges of ORs, 95% CIs and p values for the BMI-related measures associated with each reproductive disorder.
We have made the requested additions on lines 15-16. However, in lines 12-15, we summarise **30** associations (3 obesity traits x 5 reproductive disorders x 2 observational/genetic), and therefore decided against including 30 sets of ORs, 95% CIs and p values to keep within the recommended Abstract length. Similarly, lines 17-18 describe **8** separate associations (2 obesity traits x 4 reproductive disorders), so we did not expand upon all of the ORs, 95% CIs, and p values to keep the Abstract concise. These are all available in the Results.

4. Abstract: Line 25: Please avoid the use of language that implies causality (e.g. please replace “increased risk of” with “were associated with increased risk of”).
Replaced.

5. Abstract: Line 26: We suggest removing “one of the first” from the sentence.
Removed, the line now reads: “... in a systematic, large-scale, genetics-based analysis…”

6. Author summary: Line 43: Please revise to: “We found that inherited genetic variation that is associated with obesity…”
Revised, the line now reads: “... genetic variation that is associated with obesity is also associated with female reproductive disorders…”

7. Introduction: Line 59: Please clarify the sentence to indicate the direction of the relationship (for example, is increased BMI associated with increased prevalence of gynaecological conditions?)
Clarified to read “... increased body mass index (BMI) is associated with increased prevalence…”

8. Methods: Line 141-142: Prospective analysis plan. Thank you for noting that no prospective analysis plan was followed. Please state in the Methods section when each of the analyses were planned, and if/when reported analyses differed from those that were planned. For example, any changes made in the analyses, including those made in response to peer review comments, should be identified as such in

the Methods section of the paper, with rationale. If a reported analysis was performed based on an interesting but unanticipated pattern in the data, please be clear that the analysis was data-driven.

We have now added “... in response to peer review comments” or similar to each line in the Methods that describes an analysis added following peer review. Furthermore, the Methods section of the paper has been expanded to include the following rationale:

“As a data-driven investigation, the analyses upon which this paper is based were planned and designed for each of the sections independently; no overall prospective analysis plan was followed. Analyses dependent upon results from other sections, such as the mediation analysis dependent on associations from two-sample MR, are noted as such within each section.”

9. Methods: STROBE statement: Thank you for including the STROBE Checklist. We request that you please also include and report your study according to the recently published STROBE-MR checklist (Skrivankova VW, Richmond RC, Woolf BAR, Yarmolinsky J, Davies NM, Swanson SA, VanderWeele TJ, Higgins JPT, Timpson NJ, Dimou N, Langenberg C, Golub RM, Loder EW, Gallo V, Tybjaerg-Hansen A, Davey Smith G, Egger M, Richards JB. Strengthening the Reporting of Observational Studies in Epidemiology Using Mendelian Randomization: The STROBE-MR Statement. JAMA. 2021;326(16):1614-1621.)

Please add the following statement, or similar, to the Methods: "This study is reported as per the Strengthening the Reporting of Observational Studies in Epidemiology Using Mendelian Randomization (STROBE-MR) guideline (S1 Checklist)."

Checklist completed and added as supplementary information; the statement has also been added to the Methods.

10. Results: Line 280: Please report p values as p<0.001, unless there is a rationale for presenting the exact p value. Please also provide 95% CIs (here and throughout where p values are presented).
We believe it is important to report the exact p value as it allows readers to interpret the significance of the association after adjustment for multiple testing - for example, had 100 tests been performed, *P* < 0.001 no longer conveys the appropriate probability of a result as extreme as the one observed occurring due to chance. 95% CIs have been added to the text throughout.

11. Results: Line 281-283: Please report both the 95% CIs and p values for these associations.

We find that including 95% CIs and p values for each of the 10 associations reported here obscures the message of the sentence. Including the CIs and p values would have the sentence read:

“Associations with WHRadjBMI were null or lower than those for WHR (ORs and 95% CIs for WHRadjBMI v. WHR for PCOS = 1.06 (0.986 – 1.13), *P* = 0.124  v. 1.48 (1.41 – 1.55), *P* = 3.32E-26; pre-eclampsia = 1.02 (0.982 – 1.07) v. 1.13 (1.09 – 1.17), endometriosis = 1.02 (0.990 – 1.04), *P* = 0.272 v. 1.087 (1.05 – 1.10), *P* = 4.97E-09; HMB = 1.06 (1.04 – 1.07), *P* = 5.30E-13 v. 1.14 (1.12 – 1.16), *P* = 8.35E-69; and UF = 1.02 (1.01 – 1.04), *P* = 2.94E-03 v. 1.08 (1.06 – 1.09), *P* = 2.75E-23), indicating that BMI may be driving many of the associations between WHR and female reproductive diseases (Fig 1 & Fig 2, Table 2).”

We believe this is difficult to understand, and instead, we would like to point readers to Table 2, which contains the CIs and p values.

12. Results: Line 294: Please clarify in the Methods if this term was used when participants were surveyed. We suggest revising “comparatively plumper than average” to something that might be more universally clear such as “having comparatively greater adiposity than average” or similar, here and in S6 table.

Lines 110-111 in the Methods has now been clarified to state that this was indeed in the language used in the UK Biobank questionnaire, now reading: “Comparative body size at age 10 (as self-reported in a questionnaire with options: “thinner”, “plumper”, or “about average”) was used…” The text has been revised to read “having comparatively greater body size” in the Results and S6 Table.

13. Results: Line 294-303: Please report both the 95% CIs and p values for these associations.

Reported.

14. Results: Line 312-313: Please revise to “While genetically predicted BMI was also associated with increased risk of most…”

Revised.

15. Results: Line 319-320: Please revise to “The differential association of genetically predicted body fat distribution with female reproductive traits…”

Revised.

16. Results: Line 347: Please revise the reference call-out style here to [35,36].

Revised.

17. Results: Line 392-394: Please revise “effect on” to “associations with” in this sentence.

Revised.

18. Discussion: Line 419: Please avoid “one of the first” or temper statements regarding primacy with “to the best of our knowledge…” or similar.

Edited, the line now reads: “In this systematic genetics-based…”

19. Discussion: Line 420: Please avoid the use of language that implies causality (e.g. please replace “increase risk of” with “ associated with increased risk of”).

Revised.

20. Discussion: Line 421: Please clarify what is meant by “whose effects may be non-uniform” in the sentence. We suggest revising to “...a broad range of reproductive conditions, and these associations may be non-uniform across the obesity spectrum.” or similar.

Revised.

21. Discussion: Line 422-432: Please replace “effects” with “associations” in this sentence.

Replaced.

22. Discussion: Line 439: Please revise to: “with each S.D. increase in BMI associated with double the risk of preeclampsia...:”

Revised.

23. Discussion: Lines 452-453: Please revise to: “...the MR analyses indicated that each S.D. higher genetically predicted BMI was associated with an increased PCOS risk of only 13%. Similarly, 1 S.D. higher genetically predicted WHR and WHRadjBMI was associated with a 24% increase in endometriosis risk, while the observational analyses suggest associations with more modest increases in risk of 7% and 2% respectively.” or similar.

Revised.

24. Discussion: Lines 488-489: Please revise to: “Genetically predicted visceral adipose tissue mass was associated with iincreased risk of PCOS and pre-eclampsia…”

Revised.

25. Discussion: Line 502: We suggest replacing “plumper” with “having greater than average adiposity” or similar.

Replaced with “having comparatively larger body size”.

26. Discussion: Line 523-524: We suggest revising to: “Finally, genetic clustering of female reproductive conditions revealed evidence supporting common genetic causes of obesity on endometriosis, UF, and HMB, which are known to share mechanisms of development.”

Revised.

27. Discussion: Line 550: Please change “on participants” to “with participants” in this sentence.

Changed.

28. Discussion: Line 557: We suggest changing to “...obesity is associated with increased risk of female reproductive disease” or similar.

Given that our work attempts to address putative causal mechanisms by which obesity increases the risk of developing female reproductive conditions, through a genetics-based approach, we believe this sentence should indeed suggest exploring **causal** pathways via other methods, including experimental and clinical approaches. Therefore, we think it is important to maintain the implication of causality in this line.

29. Discussion: Line 558: We suggest changing “cheap” to “affordable” in this sentence.

Revised.

30. References: In reference 29 and 47, please remove the disclosure information.

Removed.

31. Table 2,Table 3, and Table 4: Please present p values as p<0.001 where applicable, unless there is rationale to present exact p values.

As explained in the response to comment 10, we believe it is necessary to provide exact p values to allow readers to interpret the significance of results, particularly when adjusted for multiple testing.

32. Table 2: Please note factors adjusted for in the observational analyses in the legend. Please also provide results from unadjusted analyses.

The legend for Table 2 and caption for Figure 1 have now been modified to include the covariates adjusted for. Results from unadjusted analyses have been included in Supplementary Table 5.

33. Figure 5: In panel A, please define the “x” and “y” axes.

Figure 5A displays UMAP plots, which are representations of the relative pairwise distance between points. The dimensions (axes) of UMAP plots are essentially arbitrary, i.e. they have no inherent meaning, similar to tSNE plots. The convention is to leave these axes unlabelled (see the UMAP guidelines here: <https://umap-learn.readthedocs.io/en/latest/plotting.html>), but we added the “x” and “y” labels upon a previous request from the editor to label the axes. We would prefer to leave these axes unlabelled, or simply as “x” and “y”.

34. Supporting Information Figures: We suggest formatting all parts of each figure/caption to fit together within 1 file.

Thank you very much for this suggestion; we attempted to format files S1 Figure and S2 Figure to include all panels in a single file. However, we found it difficult to maintain readability due to the density of information being displayed (for example, S1 Figure shows 192 associations, between 8 obesity traits x 8 female reproductive disorders x 3 MR methods). We would appreciate any guidance on how to format this figure.

35. S16 Table: There is an extra “hormones.” at the end of the Table title in the file.

Removed.

36. Supporting information Tables (for example, S12 Table): Please report p values as p<0.001 where applicable, except under circumstances where there is rationale for reporting the exact p values.

Please see rationale for reporting exact p values explained in the response to comment 10.

**Comments from Reviewers:**

Reviewer #1: The critiques have been well addressed.

Reviewer #2: The authors have adequately responded to my previous comments and I commend them on an excellent paper.

Reviewer #3: I thank the Authors for addressing my comments.
